# Sparse-Temporal Crop Mapping with Confidence-Weighted Multi-Source Supervision in Jilin Province During 2019

**DOI:** 10.3390/s26216946

**Published:** 2026-11-03

**Authors:** Lun Dong, Han Yang, Yong Zhang, Shuai Wang, Weiguang Li, Chunting Zuo

**Affiliations:** 1 Department of Mathematics and Physics, Changchun Guanghua University, Changchun 130033, China; donglun@ghu.edu.cn (L.D.); yanghan@ghu.edu.cn (H.Y.); wangshuai@ghu.edu.cn (S.W.); 2 School of Electrical Information, Changchun Guanghua University, Changchun 130033, China; liweiguang@ghu.edu.cn (W.L.); zuochunting@ghu.edu.cn (C.Z.)

**Keywords:** crop mapping, sparse-temporal observation, multiple crop maps, weak supervision, knowledge distillation, Sentinel 1 and Sentinel 2

## Abstract

Reliable crop mapping under incomplete seasonal observation is difficult because temporal evidence is sparse and public crop maps can disagree in parcel boundaries and class assignments. We investigate sparse-temporal crop mapping in Jilin Province using monthly Sentinel 1 and Sentinel 2 features acquired from June to September 2019, while treating three public crop maps as complementary supervision sources rather than independent ground truth. Their labels are harmonized into four map-derived classes and transformed into a hard pseudo target, heuristic pixel weights, soft label distributions, and agreement or conflict region codes. A full-temporal teacher then guides a sparse-temporal student through confidence-weighted losses, logit and feature distillation, and prototype regularization, allowing the same four-month interface to support different observation subsets. Under the fixed July and August setting, the STLRNet student reaches 0.792 OA, 0.742 Macro F1, and 0.596 mIoU, whereas complete June-to-September observations reach 0.821 OA. Soft pseudo labels improve OA from 0.721 to 0.751 relative to unweighted hard labels, and the student requires fewer parameters, operations, inference time, and memory than the full-temporal teacher. All metrics quantify agreement with the constructed pseudo reference, so the findings establish methodological feasibility for this Jilin 2019 case study rather than independent field accuracy or cross-region transfer.

## 1. Introduction

### 1.1. Background and Motivation

Crop mapping provides geospatial information for agricultural monitoring, crop structure analysis, and land management [1]. The increasing availability of Earth observation archives supports the use of satellite image time series to represent seasonal crop dynamics [2]. Crops with similar spectral responses at one acquisition date can become separable when their phenological trajectories are observed across a longer temporal window [3,4,5]. Optical imagery captures spectral and vegetation signals, whereas synthetic aperture radar contributes complementary sensitivity to canopy structure, surface roughness, and moisture under cloudy or otherwise unfavorable optical conditions [6,7,8]; their combination provides complementary observations for the crop mapping task considered here.

Regional crop mapping remains constrained by incomplete observations and limited reference information [9]. Cloud contamination and acquisition gaps can interrupt optical time series during the growing season [1,2]. Public mapping products may differ in their sensor inputs, production methods, spatial resolutions, and class definitions, creating inconsistencies in their boundaries and class assignments [10]. We therefore treat public crop maps as map-derived supervision sources rather than independent ground truth and evaluate how a model agrees with their harmonized reference when only part of the seasonal sequence is observed.

Our case study uses the Sparse-Temporal Label-Robust Network with monthly Sentinel 1 and Sentinel 2 feature stacks from June to September 2019 in Jilin Province. A fixed four-month interface and a month mask represent different observation subsets without changing the network input structure, while the three crop maps are harmonized into map-derived pseudo labels, heuristic pixel weights, soft label distributions, and conflict indicators. A full-temporal teacher and a sparse-temporal student are optimized with confidence-weighted losses and distillation terms; the teacher supplies complete sequence information as a methodological reference, not as independent ground truth.

### 1.2. Related Work

Multi-temporal and multi-source remote sensing studies show that seasonal observations help to separate crop types with similar instantaneous spectral signatures [2,3]. Sentinel 2 time series improve crop classification by capturing phenological variation across the growing season, while deep models learn temporal structure more directly than manually designed feature aggregation [3,4,5]. Radar time series add information about canopy structure and moisture, and optical radar fusion can therefore improve crop discrimination [6,7,8]. Related IJGI studies have examined crop identification from multi-temporal active and passive observations [11], the effect of multi-source vegetation index density on classification accuracy [12], and the fusion of Sentinel 1 data with topographic and cadastral information for crop-type-enriched land cover mapping [13]. Together, these results establish the value of richer temporal and source information and motivate examining how crop mapping behaves when observations or reference information are incomplete.

Deep representation learning has advanced crop and land cover classification by optimizing spatial and temporal features directly for dense prediction [2]. Convolutional and recurrent models learn crop representations from image time series and improve the spatial generalizability of dynamic maps [4,5]. Attention-based architectures are especially relevant because they can model dependencies among observations and assign greater weight to informative temporal evidence, with both general self-attention and remote-sensing-specific designs providing flexible sequence representations [14,15]. Sequential recurrent encoders have likewise shown that explicit temporal encoding can transform satellite image sequences into discriminative land cover representations [16]. Their performance under incomplete observations requires an explicit account of observation availability [2].

Training label reliability becomes critical when reference data come from public crop maps rather than homogeneous field surveys, because mapping products may differ in sensor inputs, production years, spatial resolution, class taxonomies, and postprocessing, creating inconsistencies when they are combined [10]. Reference quality can substantially affect supervised mapping performance, particularly when similar classes are mislabeled [17], while label hierarchies can reduce ambiguity in image time series classification [18] and knowledge distillation can transfer predictive distributions and intermediate representations from a stronger teacher to a constrained student [19,20]. These studies motivate examining sparse observation handling, multi-source map harmonization, confidence weighting, and teacher–student distillation within a common crop mapping workflow.

Four lines of recent work are especially relevant to this case study. Attention-based temporal models and dynamic mapping systems use Sentinel 2 sequences to represent crop phenology and produce early- or in-season maps [21,22,23]; missing observation studies either learn directly from incomplete sequences or jointly impute observations and classify crops [24,25]; label-efficient methods use intermediate supervision, self-supervised pretraining, temporal sample migration, or weak annotation-based pseudo labels, but generally rely on a reference source different from the multiple crop maps considered here [26,27,28,29]; and cross-regional or cross-year studies show that temporal shifts, feature distribution changes, and target-domain label scarcity can reduce transfer performance [30,31,32,33,34]. These findings motivate the present focus on sparse-temporal inputs and map-derived supervision while making clear that one region and one year cannot establish transferability across settings.

### 1.3. Research Gap and Contributions

This work asks how sparse-temporal observations interact with disagreement among map-derived supervision sources and whether model comparisons remain interpretable when the available month budget changes. The questions are therefore methodological and empirical, with no implication of regional or cross-year generalization.

We address these questions through a sparse-temporal crop mapping case study whose supervision is derived from multiple crop maps, with the following contributions:

- We design a lightweight Sparse-Temporal Label-Robust Network that preserves a fixed four-month input axis while combining month-wise spatial encoding, learnable month embeddings, masked temporal attention, and dense decoding, so that one model can process one-month, two-month, three-month, and complete observation settings without changing its interface.
- We construct confidence-weighted multi-source supervision from three public crop maps by harmonizing their target categories and deriving pixel labels, heuristic weights, soft label distributions, and conflict indicators that distinguish clean agreement, weak evidence, source disagreement, and ignored pixels during training.
- We develop a teacher–student optimization scheme that transfers complete temporal information to the sparse student through confidence-weighted losses, logit distillation, feature distillation, and prototype compactness regularization, while using full-temporal predictions as methodological guidance rather than independent labels.
- We establish an evaluation protocol spanning sparse model comparison, month sensitivity, supervision and component ablations, qualitative representation behavior, and computational efficiency, while making explicit the limits of agreement metrics derived from assembled map labels.

The central contribution is not a claim that temporal attention, confidence weighting, knowledge distillation, or prototype regularization is new in isolation. The contribution is their task-driven integration into one workflow for the joint presence of sparse seasonal observations and disagreement among multiple crop maps. The fixed temporal interface preserves month identity, the supervision construction retains explicit source conflict information, and the full-temporal teacher guides a compact sparse-temporal student. This combination links observation availability with supervision uncertainty while keeping the resulting claims within the Jilin 2019 case study.

## 2. Materials and Methods

### 2.1. Study Area, Geospatial Data, and Label Preparation

Aligned 2019 remote sensing and crop map data prepared for Jilin Province support this case study of a major agricultural region in Northeast China. Monthly Sentinel 2 optical features and Sentinel 1 radar features cover June to September, matching the main crop growth period and providing phenological evidence [3,4]. Published studies also support complementary optical and radar information for crop mapping [7,8,11].

Each month is represented by a 16-channel feature vector comprising seven Sentinel 2 bands, five vegetation indices, and four Sentinel 1 radar descriptors. NDVI, EVI, LSWI, GCVI, and NDRE encode optical vegetation dynamics, whereas VV, VH, their difference, and their empirical quotient describe complementary radar responses; all indices are used as engineered phenological or structural features rather than independent reference information.

For 2019, optical observations were obtained from the harmonized Sentinel 2 Level 2A surface reflectance collection COPERNICUS/S2_SR_HARMONIZED in Google Earth Engine. Scenes with CLOUDY_PIXEL_PERCENTAGE below 80 percent were retained. Pixels assigned to classes 3, 8, 9, 10, and 11 in the scene classification layer were masked to remove cloud shadow, medium- and high-probability cloud, cirrus, and snow or ice contamination. The seven reflectance bands were divided by 10,000 before index calculation. For each month from June to September, the available masked observations were reduced by the per-pixel median.

Let ρ denote the scaled surface reflectance. The five optical indices were computed as

(1) $$\begin{array}{cc} \mathrm{NDVI} & =\frac{\rho_{B8}-\rho_{B4}}{\rho_{B8}+\rho_{B4}},\\ \mathrm{EVI} & =2.5\frac{\rho_{B8}-\rho_{B4}}{\rho_{B8}+6\rho_{B4}-7.5\rho_{B2}+1},\\ \mathrm{LSWI} & =\frac{\rho_{B8}-\rho_{B11}}{\rho_{B8}+\rho_{B11}},\\ \mathrm{GCVI} & =\frac{\rho_{B8}}{\rho_{B3}}-1,\\ \mathrm{NDRE} & =\frac{\rho_{B8}-\rho_{B5}}{\rho_{B8}+\rho_{B5}}. \end{array}$$

Radar observations were obtained from the Sentinel 1 Level 1 ground range detected collection COPERNICUS/S1_GRD after selecting interferometric wide-swath scenes containing both VV and VH. Earth Engine applied orbit file updates, border noise removal, thermal noise removal, radiometric calibration, terrain correction, and conversion to decibels before these data entered the monthly composite. No orbit direction restriction or additional speckle filter was applied in the project preprocessing script. Monthly VV and VH were obtained by the per-pixel median. The two derived radar descriptors were then computed as VVminusVH=VV−VH and $\mathrm{VVdivVH}=\frac{\mathrm{VV}}{\mathrm{VH}}$. Both descriptors were therefore calculated from decibel values. The second descriptor is an empirical quotient of decibel values rather than a linear power ratio.

All monthly features were exported at a nominal spatial scale of 10 m. The stored feature shards use EPSG 4326 with an angular pixel spacing of approximately $8.98315\times 10^{-5}$ degrees. During local preparation, each exported feature shard was retained as the reference grid, and monthly continuous features were stacked by direct window reads without further reprojection or interpolation.

The channel order within every month is B2, B3, B4, B5, B8, B11, B12, NDVI, EVI, LSWI, GCVI, NDRE, VV, VH, VV minus VH, and VV divided by VH. Nonfinite feature values are replaced by zero after reading. Every channel is then standardized with the mean and standard deviation calculated only from training patches, and the same statistics are applied to validation and test patches.

Three public crop maps provide the supervision used for cross-map agreement analysis. Majorcrop [35] is treated as the primary source, Crop10m [36] as a second fine-resolution source, and NEcrops30m [37] as coarse auxiliary evidence; the local configuration identifies all three files as 2019 products. Majorcrop and Crop10m have native pixels of approximately 10 m, whereas NEcrops30m has approximately 30 m pixels, and all three categorical map rasters are reprojected to the feature grid by nearest-neighbor resampling. Consequently, the 30 m product contributes weak coarse-scale evidence rather than independent 10 m ground truth.

All three products were clipped to the common Jilin study extent before patch extraction. The local input configuration identifies the three products as 2019 layers, but comparable independent field or parcel accuracy estimates for this clipped extent have not been established here. We therefore report their year, native grid, class coding, and supervision role in Table 1, without inferring source accuracy from the model results. The *other* class is a valid residual crop-map category that participates in pseudo-label construction and evaluation; the *ignore* class denotes no data, unmapped values, and excluded pixels that do not participate in loss or metric computation.

**Figure 1:**
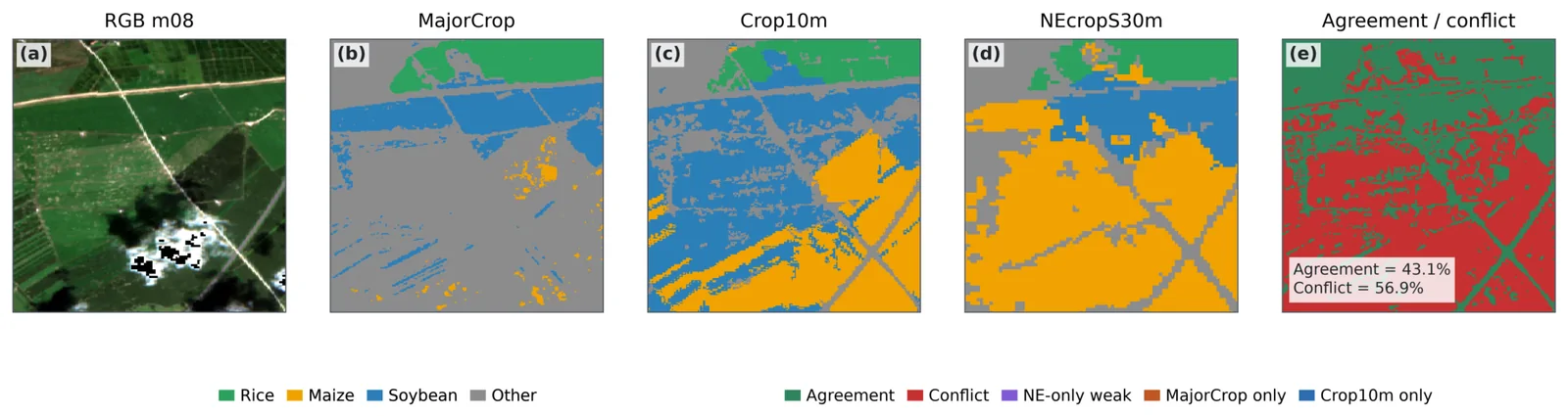
Example of multi-source label agreement and conflict regions generated from the harmonized crop maps. The visualization separates pixels with consistent labels from pixels with source-dependent conflicts, thereby indicating where confidence-aware supervision is required.

Figure 1 illustrates the spatial mask that separates agreement between the two principal sources, source conflict, weak evidence, and ignored regions. Agreement pixels receive support for the same class, whereas conflict pixels retain locations where the maps disagree; the region code is not introduced as an additional land cover class but instead controls pseudo-label confidence, soft supervision, and conflict region analysis, thereby enabling separate reporting of overall agreement and agreement in the most uncertain areas.

Aligned rasters are divided into nonoverlapping patches of 256 by 256 pixels with a stride of 256 pixels, and a candidate is retained only when at least 30% of its pixels have valid map-derived pseudo labels, at least 10% belong to the three explicit crop classes, and the mean heuristic label weight is no lower than 0.05. These criteria describe map coverage rather than ground truth quality, yielding 21,952 training patches, 5421 validation patches, and 5603 test patches.

Patches are assigned to training, validation, and test subsets by a grid identifier. A deterministic SHA1 hash combines random seed 42 with each grid identifier and maps the grid to a stable value between zero and one. Values below 0.60 enter the training subset, values from 0.60 to 0.80 enter the validation subset, and the remaining values enter the test subset. Every patch from one grid inherits the same assignment. No geographic buffer is applied, and the same split lists are used for teacher training, student training, model selection, and all comparison models.

The resulting training, validation, and test subsets contain 21,952, 5421, and 5603 patches. They contain 48, 13, and 11 unique grid identifiers and 12, 9, and 8 unique tile identifiers. Grid identifiers and patch footprints do not repeat across subsets. Tile identifiers can span several grids, so 8 tile identifiers are shared by training and validation, 7 by training and test, and 5 by validation and test. The selected patch footprints have no positive area intersection across subsets, but their coverage boundaries can touch. The reported metrics therefore characterize this fixed grid assignment without a geographic separation buffer.

### 2.2. Problem Formulation and Sparse-Temporal Observation

Let T={6,7,8,9} denote the four available months, with T=4, C=16, H=W=256, and K=4 crop classes, so that each sample is represented as

(2) $$X\in R^{T\times C\times H\times W},\;Y\in {\left\{0,\ldots ,K-1,255\right\}}^{H\times W},$$

where 255 denotes the ignore index used during loss computation, and sparse-temporal availability is encoded by the vector

(3) $$m=\left[m_{1},m_{2},m_{3},m_{4}\right],\;m_{t}\in \left\{0,1\right\},\;\sum_{t=1}^{T}m_{t}>0.$$

A fixed four-month tensor is supplied to the model, with unavailable observations suppressed by the corresponding mask entries,

(4) $$X_{t}^{\mathrm{sparse}}=m_{t}X_{t}.$$

All four months are visible during teacher training, giving $m^{T}=\left[1,1,1,1\right]$, whereas the student uses July and August in the main comparison. Month sensitivity follows the configurations in Table 4; throughout these settings, the fixed temporal axis preserves month identity and availability without changing the model interface.

Previous studies address missing observations through direct learning from incomplete Sentinel 2 sequences or joint imputation and crop classification, demonstrating that cloud-related gaps can be handled without assuming that every acquisition is recoverable [24,25]. Because these findings do not establish that a month mask reproduces a fully irregular observation process, the present study treats month selection as an explicit experimental factor and distinguishes masked aggregation from imputation-based approaches.

**Figure 2:**
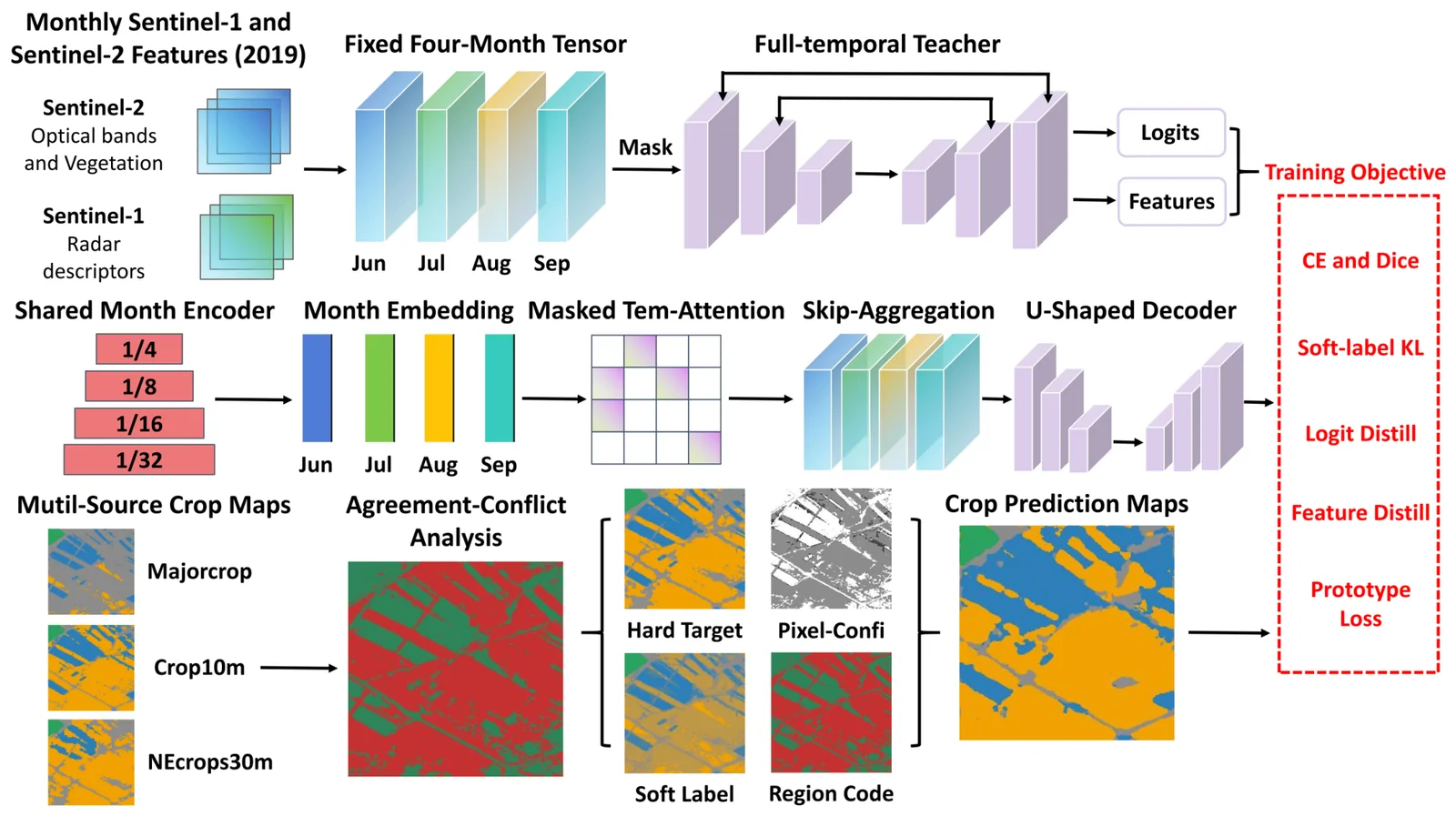
Overview of the proposed STLRNet framework. Multi-source crop maps provide confidence-aware supervision, while the full-temporal teacher transfers complete sequence information to the sparse-temporal student.

Figure 2 summarizes the supervision workflow, in which three crop maps are harmonized and their agreement patterns are transformed into a hard target, a heuristic pixel weight, a soft label distribution, and a region code. These outputs provide complementary signals for teacher and student training, while the region code retains the distinction between reliable agreement and source conflict.

**Figure 3:**
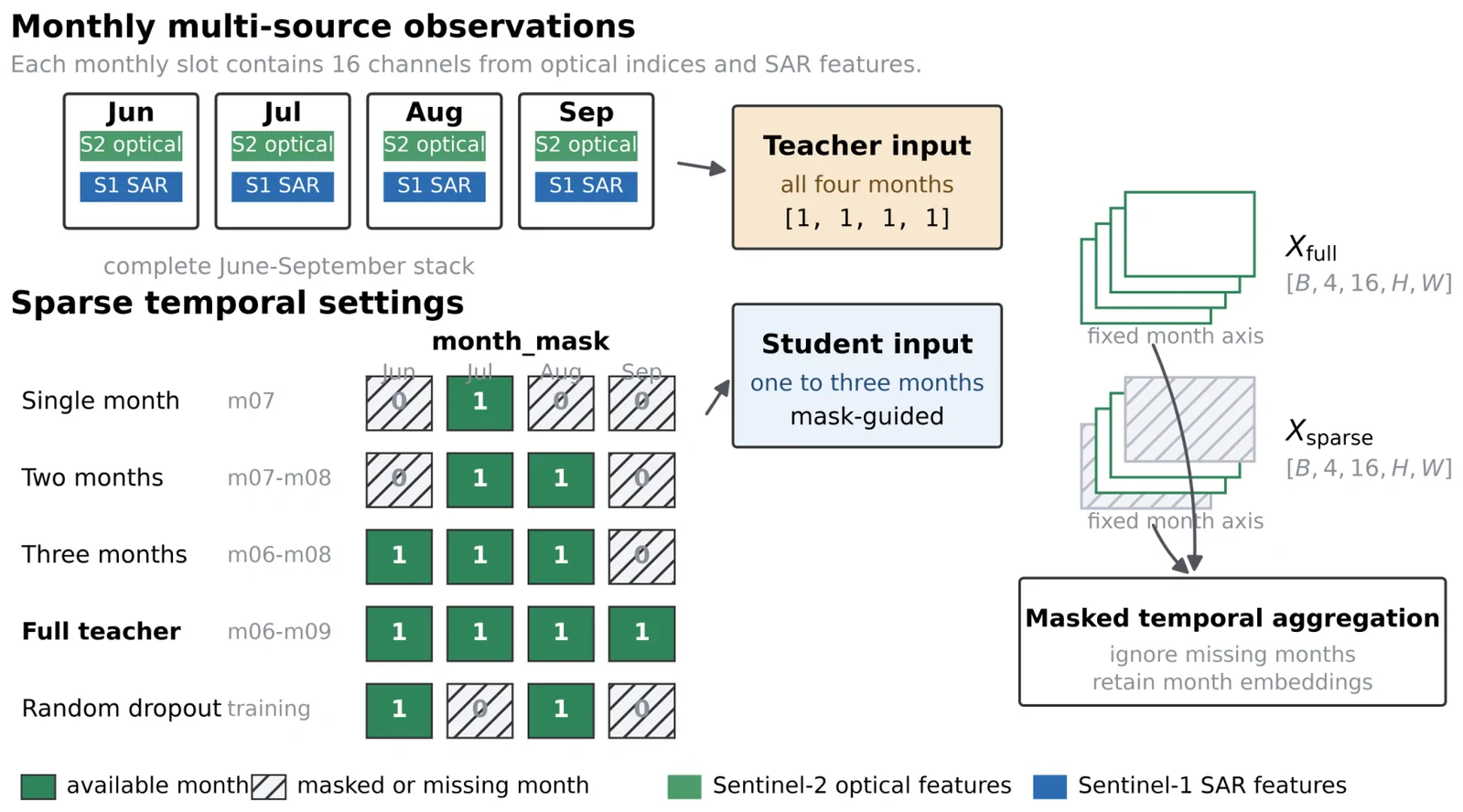
Sparse-temporal input representation. The four-month axis is retained for all samples, while the month mask indicates which observations are available to the student model.

Figure 3 illustrates the sparse-temporal setting, where unavailable months are set to zero and excluded from temporal attention rather than removed from the tensor. Retaining a fixed four-month axis preserves the position of each observation and permits direct comparison of July and August with shorter or longer combinations, thereby supporting consistent data loading, encoder sharing, and distillation; however, all four tensor positions are still processed before masking.

### 2.3. Multiple Source Pseudo-Label Construction

Each patch is associated with four supervision maps: a map-derived hard pseudo target, a heuristic pixel weight, a soft label distribution, and a region code. Let $y_{i}^{M}$, $y_{i}^{C}$, and $y_{i}^{N}$ denote the harmonized Majorcrop, Crop10m, and NEcrops30m labels at pixel *i*, with values in {1,2,3,4} considered valid; weighted voting across the three sources then defines the soft label distribution

(5) $$q_{i,k}=\frac{\alpha_{M}1\left\{y_{i}^{M}=k\right\}+\alpha_{C}1\left\{y_{i}^{C}=k\right\}+\alpha_{N}1\left\{y_{i}^{N}=k\right\}}{\sum_{j=1}^{K}\alpha_{M}1\left\{y_{i}^{M}=j\right\}+\alpha_{C}1\left\{y_{i}^{C}=j\right\}+\alpha_{N}1\left\{y_{i}^{N}=j\right\}+\varepsilon },$$

where $\alpha_{M}=0.50$, $\alpha_{C}=0.35$, and $\alpha_{N}=0.15$. These coefficients and the region weights are heuristic priors that have not been calibrated with independent field labels. Agreement between Majorcrop and Crop10m receives the shared class and a weight of 1.00; pixels represented only by Majorcrop, Crop10m, or NEcrops30m receive weights of 0.70, 0.60, and 0.30, respectively. When the two principal maps conflict, NEcrops30m is used only to support one of the competing classes, giving supported conflicts a weight of 0.55, whereas unsupported conflicts retain the Majorcrop label with a weight of 0.25. Pixels without any valid source label receive zero weight and are ignored, so all reported weights should be interpreted as design choices rather than calibrated probabilities.

For PyTorch optimization, valid class values are converted to zero-based indices:

(6) $$Y_{i}=\left\{\begin{array}{cc} {\tilde{y}}_{i}-1, & {\tilde{y}}_{i}\in \left\{1,2,3,4\right\}\;\mathrm{and}\;w_{i}>0,\\ 255, & \mathrm{otherwise}. \end{array}\right.$$

This construction expresses disagreement among public crop maps as heuristic supervision strength instead of discarding every inconsistent pixel, while retaining the region codes for conflict-specific analysis. Because targets in conflict regions are selected from the same map sources used to define those regions, the resulting metrics quantify internal pseudo-reference agreement rather than independently validated robustness.

### 2.4. Sparse-Temporal Label-Robust Network Architecture

STLRNet uses a lightweight encoder–attention decoder architecture in which a shared spatial encoder processes every monthly feature image through an initial convolutional block and depthwise separable blocks at four spatial scales. Denoting this monthly encoder by $E_{\theta }$, its multiscale outputs are

(7) $$S_{t}^{1},S_{t}^{2},S_{t}^{3},S_{t}^{4}=E_{\theta }\left(X_{t}^{\mathrm{sparse}}\right),\;t\in T.$$

When month embeddings are enabled, each bottleneck feature is augmented with a learnable vector broadcast over its spatial dimensions,

(8) $$B_{t}=S_{t}^{4}+e_{t}.$$

The model computes a spatially resolved attention score for each month as

(9) $$a_{t,u,v}=g_{\phi }\left(B_{t,:,u,v}\right),$$

where *u* and *v* index spatial location and $g_{\phi }$ maps the channel vector at a spatial location to an unnormalized month logit. Applying the month mask before attention normalization excludes unavailable observations from aggregation, although the encoder and scoring network still process all four tensor positions, so masking alone neither skips their computation nor removes their influence on batch normalization.

(10) $$\pi_{t,u,v}=\frac{m_{t}\exp a_{t,u,v}}{\sum_{\tau =1}^{T}m_{\tau }\exp a_{\tau ,u,v}+\varepsilon }.$$

Normalized attention weights produce the aggregated bottleneck feature

(11) $$F_{:,u,v}=\sum_{t=1}^{T}\pi_{t,u,v}B_{t,:,u,v}.$$

The decoder skip features are likewise obtained by masked averaging,

(12) $${\bar{S}}^{l}=\frac{\sum_{t=1}^{T}m_{t}S_{t}^{l}}{\sum_{t=1}^{T}m_{t}+\varepsilon },\;l\in \left\{1,2,3\right\}.$$

A decoder then upsamples the bottleneck representation and combines it with the masked skip features in a U-shaped architecture [38]. The classifier $h_{\psi }$ maps the decoded features to one logit map per crop class, producing four class logits,

(13) $$Z=h_{\psi }\left(D_{\eta }\left(F,{\bar{S}}^{3},{\bar{S}}^{2},{\bar{S}}^{1}\right)\right),\;Z\in R^{K\times H\times W}.$$

The network returns both class logits and the bottleneck representation. When teacher and student channel widths differ, feature distillation aligns them by truncating to the smaller channel count, an implementation choice that must be considered when interpreting the feature transfer results.

The student uses a base width of 24 channels. Its four encoder stages contain 24, 48, 96, and 192 channels, and its three decoder stages contain 96, 48, and 24 channels. The teacher uses a base width of 32 channels, encoder widths of 32, 64, 128, and 256 channels, and decoder widths of 128, 64, and 32 channels. Both networks use a dropout rate of 0.10 and a learned month embedding at the bottleneck width.

Feature distillation uses the bottleneck tensors returned by the frozen teacher and the student. When their spatial resolutions differ, the teacher tensor is resized to the student resolution by bilinear interpolation. Both tensors then retain the first $C_{a}=\min\left\{C_{S},C_{T}\right\}$ channels, where $C_{S}$ and $C_{T}$ are the student and teacher channel counts. The feature loss is the mean squared error over all retained channels and spatial positions,

(14) $$L_{\mathrm{feat}}=\frac{1}{BC_{a}H_{S}W_{S}}\sum_{b,c,u,v}{∥F_{b,c,u,v}^{S}-{\tilde{F}}_{b,c,u,v}^{T}∥}_{2}^{2},$$

where ${\tilde{F}}^{T}$ denotes the resized and truncated teacher tensor. Gradients do not pass through this teacher tensor. Channel truncation provides deterministic alignment without additional trainable parameters, but it can discard teacher information and channel order does not guarantee semantic correspondence. The present ablation supports the implemented alignment choice only.

### 2.5. Learning Objective for Teacher–Student Training

Training combines heuristic weighted pseudo-label supervision with teacher regularization. Inverse-square-root class weights computed from the training split address class imbalance, while pixel weights modulate the contributions of agreement, weak evidence, and conflict regions. The segmentation term comprises weighted cross-entropy and weighted Dice loss [39], the soft-target term uses the multi-source distribution $q_{i,k}$, and the full-temporal teacher supplies logit and bottleneck feature distillation [19,20]. Together, these losses constrain consistency with map-derived supervision and teacher predictions but cannot establish independent label accuracy.

Combining these terms gives the total objective

(15) $$L=\lambda_{\mathrm{ce}}L_{\mathrm{ce}}+\lambda_{\mathrm{dice}}L_{\mathrm{dice}}+\lambda_{\mathrm{soft}}L_{\mathrm{soft}}+\lambda_{\mathrm{kd}}L_{\mathrm{kd}}+\lambda_{\mathrm{feat}}L_{\mathrm{feat}}+\lambda_{\mathrm{proto}}L_{\mathrm{proto}}.$$

The λ terms weighted cross-entropy, Dice, soft target, logit distillation, feature distillation, and prototype compactness losses in the order shown in the objective. Student training uses $\lambda_{\mathrm{ce}}=1.0$, $\lambda_{\mathrm{dice}}=0.5$, $\lambda_{\mathrm{soft}}=0.2$, $\lambda_{\mathrm{kd}}=0.5$, $\lambda_{\mathrm{feat}}=0.1$, $\lambda_{\mathrm{proto}}=0.1$, and a distillation temperature of 2.0. These heuristic settings remain fixed throughout the case study; all four months are visible when training the teacher, whose frozen checkpoint subsequently provides student distillation targets.

Prototype compactness is computed from the student bottleneck rather than from class logits. Hard pseudo targets and heuristic pixel weights are resized to the bottleneck resolution by nearest-neighbor sampling, and each bottleneck vector is normalized along the channel dimension. For every class present in a mini-batch, the class prototype is the confidence-weighted feature mean,

(16) $$p_{k}=\frac{\sum_{i}w_{i}1_{y_{i}=k}f_{i}}{\sum_{i}w_{i}1_{y_{i}=k}+\varepsilon }.$$

The compactness loss averages the squared Euclidean distance from valid student features to their corresponding class prototype and then averages across classes present in the mini-batch. A class with no valid feature is omitted. The prototypes are recomputed for each mini-batch, no persistent prototype bank is maintained, and this loss is used only during training. Gradients pass through the student features and their class means. Thus, logit distillation constrains output distributions, feature distillation transfers an intermediate teacher representation, and prototype compactness organizes the student representation around its batch-specific class centers.

### 2.6. Experimental Design and Implementation Details

All models are implemented in PyTorch and trained with normalized features and map-derived pseudo labels, using channel statistics computed only from the fixed-grid-based training split. Spatial flips and rotations provide geometric regularization without adding independent information. For the main comparison, every model receives July and August observations under random seed 42; the student uses a batch size of 16, at most 120 epochs, early stopping after 15 epochs without improvement, mixed precision when CUDA is available, and gradient clipping at a norm of 1.0. AdamW optimization uses a learning rate and weight decay of $10^{-4}$ [40]. All reported values are single-run results under random seed 42.

Validation Macro F1 is the checkpoint selection criterion. The checkpoint with the highest validation Macro F1 is retained for test evaluation, and training stops after 15 validation epochs without improvement in this criterion. Test metrics are reported only from the retained checkpoint. This rule is shared across the comparison models and does not provide uncertainty estimates across independent runs.

The full-temporal teacher is trained with the complete June-to-September sequence and agreement between the two principal sources, so it functions as a temporal reference rather than a directly comparable sparse input model. The main student comparison uses July and August, while Table 4 evaluates other month combinations and Tables 6 and 7 examine label weighting, soft supervision, distillation, and compactness.

The main comparison includes U-Net, DeepLabV3+, TempCNN, and a Transformer baseline [14,38,41]. Every baseline and the STLRNet student use July and August, the same grid-based split, label protocol, checkpoint rule, and random seed 42; month sensitivity experiments are reported separately because they examine observation availability rather than architectural differences.

Although every comparison model receives the same July and August observations, their temporal processing differs. U-Net and DeepLabV3+ concatenate the two 16-channel feature blocks into a 32-channel spatial input and therefore retain neither an explicit month dimension nor temporal attention. TempCNN preserves the two positions, projects each monthly block into a shared embedding, and applies three-dimensional convolutions along the temporal dimension, whereas the Transformer baseline combines a shared monthly spatial encoder with masked temporal attention and a month embedding. STLRNet retains the fixed June-to-September interface and represents the July and August setting by m=[0,1,1,0], preserving both month identity and availability.

Table 2 shows that common input months, data splits, pseudo-label construction, and checkpoint rules do not imply identical training information. The selected baselines do not receive teacher outputs, whereas the STLRNet student receives complete sequence logits and bottleneck features and also uses prototype compactness. Table 3 therefore compares complete training systems under a common evaluation protocol. Its differences combine architecture, teacher information, and regularization and cannot be attributed entirely to network architecture. The component ablations remove individual terms but do not constitute a configuration that removes all teacher information while preserving every other STLRNet choice.

Evaluation uses the agreement metrics defined in Section 2.7, with overall and conflict region results reported separately.

### 2.7. Evaluation Metrics

We evaluate predictions against the constructed map-derived pseudo reference using overall accuracy (OA), average accuracy (AA), Cohen’s Kappa, Macro F1, and mean intersection over union (mIoU). Class-wise precision, recall, F1, and IoU describe differences among rice, maize, soybean, and the other class. Let $n_{kl}$ be the number of evaluated pixels whose reference class is *k* and predicted class is *l*, where k,l∈{0,…,K−1} and K=4. The reference support is $n_{k+}=\sum_{l=0}^{K-1}n_{kl}$, the prediction count is $n_{+k}=\sum_{l=0}^{K-1}n_{lk}$, and the total number of evaluated pixels is $N=\sum_{k=0}^{K-1}n_{k+}$. Pixels with the ignore index 255 are excluded, while the other class remains a valid evaluation category. For each class, true positives, false positives, and false negatives are

(17) $${\mathrm{TP}}_{k}=n_{kk},\;{\mathrm{FP}}_{k}=n_{+k}-n_{kk},\;{\mathrm{FN}}_{k}=n_{k+}-n_{kk}.$$

Precision measures the fraction of predictions assigned to a class that agree with the reference, whereas recall measures the fraction of reference pixels of that class recovered by the model:

(18) $$P_{k}=\frac{{\mathrm{TP}}_{k}}{{\mathrm{TP}}_{k}+{\mathrm{FP}}_{k}},\;R_{k}=\frac{{\mathrm{TP}}_{k}}{{\mathrm{TP}}_{k}+{\mathrm{FN}}_{k}}.$$

F1 combines precision and recall through their harmonic mean, while IoU measures the overlap between the predicted and reference pixel sets for a class:

(19) $$\begin{array}{cc} F1_{k} & =\frac{2P_{k}R_{k}}{P_{k}+R_{k}}=\frac{2{\mathrm{TP}}_{k}}{2{\mathrm{TP}}_{k}+{\mathrm{FP}}_{k}+{\mathrm{FN}}_{k}},\\ {\mathrm{IoU}}_{k} & =\frac{{\mathrm{TP}}_{k}}{{\mathrm{TP}}_{k}+{\mathrm{FP}}_{k}+{\mathrm{FN}}_{k}}. \end{array}$$

For the same class and evaluated pixel set, these definitions imply ${\mathrm{IoU}}_{k}=F1_{k}/\left(2-F1_{k}\right)$.

OA is the proportion of all evaluated pixels that agree with the reference. AA is the arithmetic mean of class recalls and gives each class equal weight:

(20) $$\mathrm{OA}=\frac{\sum_{k=0}^{K-1}n_{kk}}{N},\;\mathrm{AA}=\frac{1}{K}\sum_{k=0}^{K-1}R_{k}.$$

Cohen’s Kappa adjusts observed agreement for the agreement expected from the reference and prediction class frequencies. With observed agreement $p_{o}=\mathrm{OA}$, it is defined as

(21) $$p_{e}=\frac{1}{N^{2}}\sum_{k=0}^{K-1}n_{k+}n_{+k},\;\kappa =\frac{p_{o}-p_{e}}{1-p_{e}}.$$

Macro F1 and mIoU summarize class-wise performance using equal weights across the four categories:

(22) $$\mathrm{Macro}\;F1=\frac{1}{K}\sum_{k=0}^{K-1}F1_{k},\;\mathrm{mIoU}=\frac{1}{K}\sum_{k=0}^{K-1}{\mathrm{IoU}}_{k}.$$

The class-wise F1–IoU conversion is nonlinear, so applying it directly to Macro F1 does not generally yield mIoU. Conflict OA and conflict Macro F1 use the same definitions after restricting the evaluated pixels to the conflict region mask. All agreement metrics compare predictions with the constructed pseudo reference; their interpretation therefore remains distinct from accuracy established using independent field or parcel observations.

Metric computation accumulates one confusion matrix from every valid pixel in every test patch before any reported metric is calculated. Metrics are not calculated separately for each patch and then averaged. Pixels with reference value 255 are removed before accumulation, while rice, maize, soybean, and the other class all remain in the matrix and in every macro average. A class with no reference or predicted pixels receives zero for any component with a zero denominator and remains part of the four-class average. Overall and class-wise metrics use the same test mask, class set, retained checkpoint, and prediction set. Heuristic pixel weights used during training do not contribute to metric accumulation.

## 3. Results

All reported results quantify agreement with the constructed map-derived pseudo reference. Unless otherwise specified, OA, AA, Kappa, Macro F1, and mIoU use the same grid-based test split and label construction; interpretation is confined to Jilin Province in 2019 because no independent field or parcel reference exists.

### 3.1. Main Model Comparison

Among the sparse models in Table 3, the STLRNet student achieves the strongest agreement, with 0.792 OA, 0.772 AA, 0.647 Kappa, 0.742 Macro F1, and 0.596 mIoU. The Transformer is the best baseline, while U-Net, DeepLabV3+, and TempCNN remain lower on every metric. Because the full-temporal teacher uses four months and a cleaner supervision subset, it is retained as a methodological reference rather than included as an equivalent competitor; subsequent tables therefore isolate month availability, supervision design, and network components.

Agreement increases consistently with month availability in Table 4. July yields 0.682 OA and 0.567 mIoU, while August yields 0.697 OA and 0.571 mIoU. Their combination yields 0.792 OA, 0.772 AA, 0.742 Macro F1, and 0.596 mIoU. June to August reaches 0.807 OA, 0.786 AA, 0.774 Macro F1, and 0.633 mIoU; July to September reaches 0.813 OA, 0.803 AA, 0.792 Macro F1, and 0.657 mIoU; and the complete June-to-September sequence reaches 0.821 OA, 0.814 AA, 0.802 Macro F1, and 0.671 mIoU. This pattern is compatible with richer phenological context, although it is observed within one region, year, split, and pseudo reference.

Class-wise results in Table 5 reveal differences across categories under the July and August setting. Rice has the highest recall (0.941), with precision, F1, and IoU of 0.617, 0.745, and 0.594. Maize has the highest precision (0.896), F1 (0.816), and IoU (0.689), with recall of 0.749. Soybean has the lowest precision (0.548), recall (0.673), F1 (0.604), and IoU (0.433). The other class has precision, recall, F1, and IoU values of 0.895, 0.725, 0.801, and 0.668. All values describe agreement with the constructed pseudo reference rather than field accuracy.

### 3.2. Supervision and Label Ablation

The supervision settings differ in both target construction and evaluation scope. The Majorcrop-only, Crop10m-only, and NEcrops30m-only configurations use the selected map classes as both the training target and the evaluation reference, retain only pixels with a valid class in that source, and otherwise keep the common student terms. The high-confidence configuration uses the class shared by Majorcrop and Crop10m, evaluates agreement pixels only, and excludes ambiguous and conflict pixels. Their scores therefore involve different references or pixel populations and are not directly comparable with results obtained over the complete constructed reference.

For the broader constructed reference population, the hard label configuration trains on the constructed hard pseudo target, evaluates all valid constructed reference pixels, assigns unit pixel weights, and removes the soft-target contribution. The weighted configuration keeps the same hard-target and evaluation reference but applies heuristic pixel weights while retaining the shared soft loss path, whereas the soft label configuration additionally uses the constructed soft distribution. The complete STLRNet student uses the full constructed supervision over the same valid population together with the selected distillation and compactness terms. Direct progression claims are therefore limited to the hard, weighted, and soft settings, while the complete student remains a system-level result.

Table 6 separates the effects of source choice, evaluation scope, and supervision formulation. Majorcrop produces the strongest single-source result with 0.821 OA, 0.812 Macro F1, and 0.694 mIoU, followed by Crop10m (0.806, 0.787, and 0.668) and NEcrops30m (0.793, 0.767, and 0.626). Restricting evaluation to high-confidence regions yields 0.824 OA, 0.815 Macro F1, and 0.712 mIoU, but this row is not directly comparable with full-region evaluation because ambiguous pixels are excluded. Among settings retaining the broader supervision population, unweighted hard labels yield 0.721 OA, 0.693 Macro F1, and 0.532 mIoU; weighting raises these values to 0.732, 0.706, and 0.547, while soft pseudo labels reach 0.751, 0.718, and 0.562. Conflict region agreement also increases across these settings: OA and Macro F1 change from 0.617 and 0.581 for hard labels, to 0.623 and 0.592 with weighting, and to 0.638 and 0.608 with soft labels. The complete STLRNet student reaches 0.792 OA, 0.742 Macro F1, and 0.596 mIoU, together with 0.674 conflict OA and 0.635 conflict Macro F1. Because this row combines the selected supervision components with temporal masking, teacher guidance, and network regularization, its gain cannot be attributed to one label operation alone. Moreover, weighted and soft pseudo-label settings share the same soft loss path in the implementation and should be interpreted as controlled configurations rather than fully independent causal ablations; the month mask, data split, random seed, and checkpoint rule remain fixed as the supervision input changes.

The Majorcrop-only value can be higher because the model is evaluated against the same source that defines its target and because its valid pixel population differs from the complete constructed reference. It does not demonstrate that Majorcrop supervision is superior to the complete method on one common evaluation target. Likewise, the high-confidence result reflects an easier restricted evaluation after conflict and ambiguous pixels are removed.

**Figure 4:**
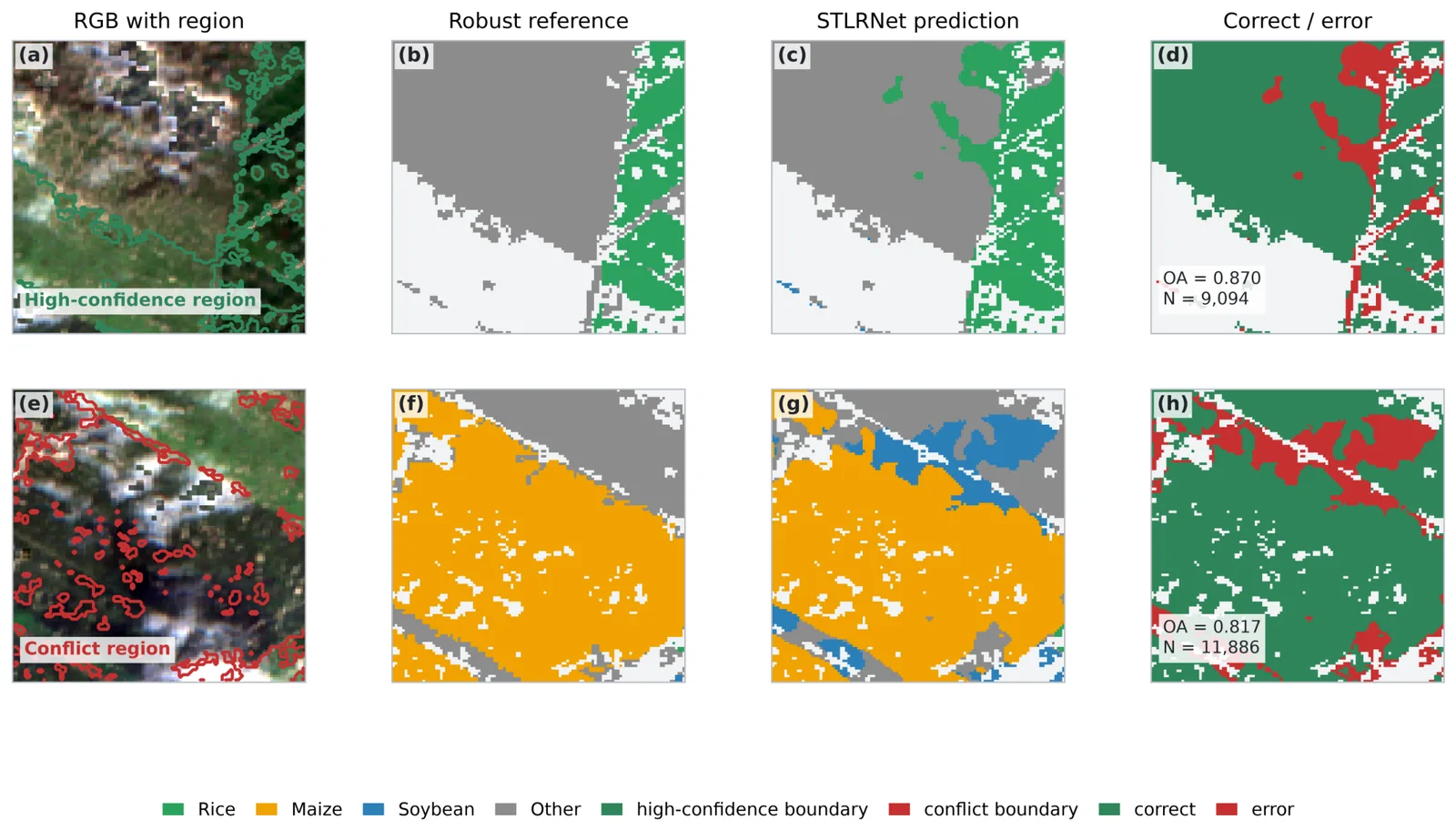
Comparison of label agreement regions and label conflict regions under the supervision settings. The maps describe spatial agreement with the constructed pseudo reference.

Figure 4 provides spatial context for the label ablation: high-confidence regions form more coherent supervision areas, whereas conflict regions concentrate disagreement among the source maps. Such clustering can help to explain the higher scores obtained under restricted evaluation, but it neither establishes independent classification accuracy nor identifies any single map as correct.

Component ablation in Table 7 shows that the complete student obtains 0.792 OA, 0.772 AA, 0.647 Kappa, 0.742 Macro F1, and 0.596 mIoU. Removing logit distillation reduces OA, Kappa, and mIoU to 0.741, 0.629, and 0.541, respectively, while removing feature distillation produces the largest decline, to 0.712 OA, 0.613 Kappa, and 0.498 mIoU. Omitting compactness loss has a smaller effect, yielding 0.748 OA, 0.632 Kappa, and 0.554 mIoU, and matching the teacher width reaches 0.763 OA, 0.637 Kappa, and 0.564 mIoU without recovering the complete model. Channel width alone therefore does not explain the observed differences, although interactions among optimization terms may still influence the representation because the ablations share the same month setting, supervision protocol, split, seed, and checkpoint rule rather than forming an exhaustive factorial design.

### 3.3. Computational Efficiency and Evaluation Scope

Resource measurements in Table 8 show that the student reduces the parameter count from 780,549 to 443,141, GFLOPs from 19.106 to 11.309, inference time from 5.82 to 4.61 ms, and memory from 5091.5 to 3946.3 MB. This smaller exported model trades agreement for efficiency, as OA changes from 0.883 to 0.792 and Macro F1 from 0.875 to 0.742. Because the encoder still processes all four temporal positions before masked aggregation, the GFLOP reduction reflects channel width and the complete implementation rather than only the cost of inactive months.

Efficiency was measured on one NVIDIA A40 GPU with PyTorch 2.5.1 and CUDA 12.1. Teacher and student inputs use four temporal slots, 16 channels per slot, a spatial size of 256×256 pixels, and float32 inference. Latency uses a batch size of 32 and the complete test loader. CUDA synchronization is applied immediately before and after each model forward pass, the synchronized forward time is summed over the loader, and the total is divided by the number of patches. Data loading, metric calculation, and file writing are outside the timed interval. GFLOPs count convolution and linear multiplication and addition as two operations for one patch. Peak allocated memory uses a synthetic batch of 32 after one preliminary forward pass and a reset of the CUDA peak memory counter. The table reports exported model inference cost only. It does not include teacher training, student distillation training, data preparation, or the combined cost of the complete training workflow.

**Figure 5:**
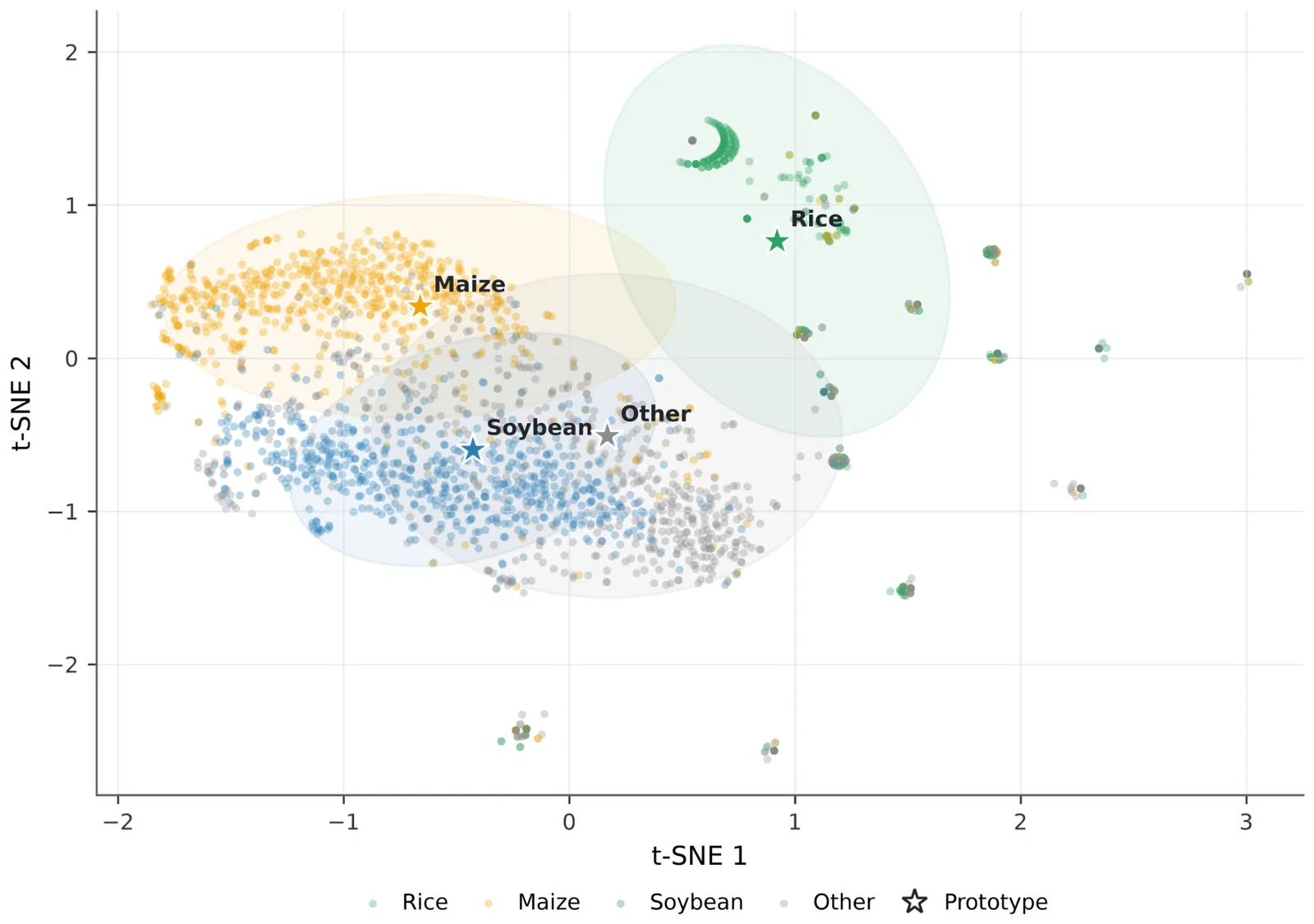
t-SNE visualization of normalized bottleneck features from the sparse-temporal student with July and August input. Class prototypes are represented by embedded feature centroids.

**Figure 6:**
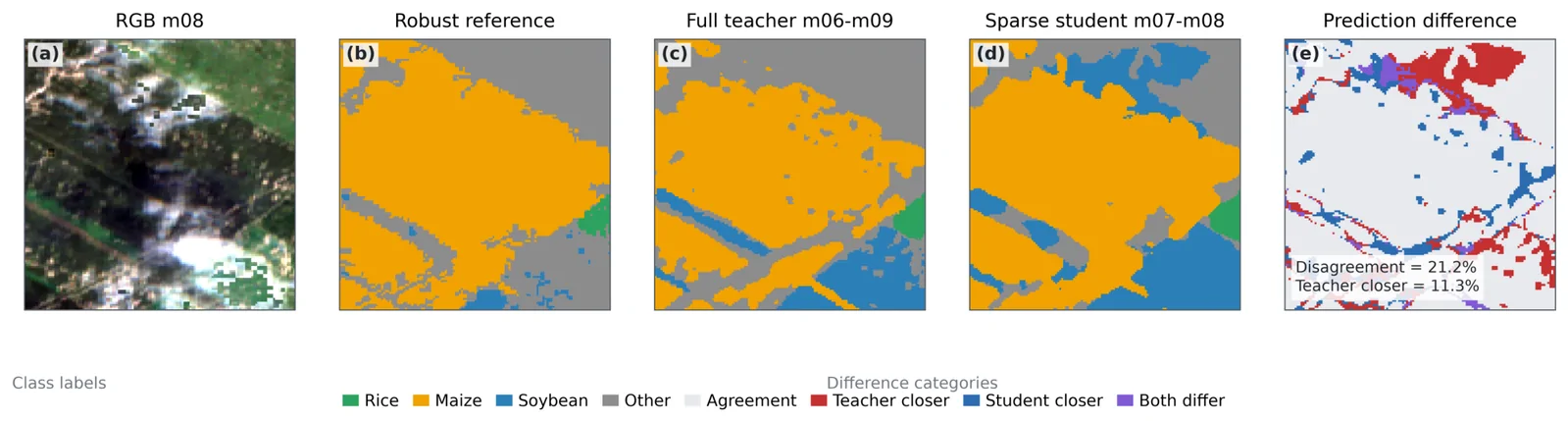
Spatial prediction difference between the sparse-temporal student and the full-temporal teacher for a representative crop patch. Difference categories indicate whether the teacher, the student, or both models deviate from the reference label.

Figure 5 and Figure 6 complement the numerical results by showing feature organization under the July and August setting and localizing student–teacher disagreement within a representative patch. Concentrated differences may indicate locations where sparse observations or ambiguous pseudo labels challenge the student, although these visualizations cannot quantify calibration, independent field accuracy, or broader transfer.

Independent field or parcel reference data are unavailable, so the numerical metrics, qualitative maps, and teacher–student comparisons all describe agreement with the constructed pseudo reference. This distinction is especially important for source-restricted settings and high-confidence regions, whose higher values partly reflect the removal of ambiguous supervision pixels.

## 4. Discussion

Results from the Jilin 2019 case study indicate that sparse-temporal crop mapping can maintain substantial agreement with a constructed pseudo reference while using fewer observations than the full-temporal teacher. Evidence from the architecture comparison, month sensitivity analysis, supervision and component ablations, and resource profiling supports the methodological feasibility of this design, although interpretation remains bounded by the reference construction, grid identifier split, single region and year, and absence of independent field or parcel labels.

Representative studies provide supporting evidence under different protocols rather than directly comparable rankings. Experiments in Hengshui, China, used dense Sentinel 2 sequences with 43.5 percent missing sample values and crop reference samples; three of five tested models exceeded 85 percent overall accuracy on unfilled sequences [24]. A 2016 study in Shawan County, China, used ten Sentinel 2A dates from April to October, including three dates partly affected by cloud, and jointly performed imputation and pixel-wise crop classification; overall accuracy improved by as much as 4.2 percentage points over the reported BiLSTM comparison [25]. Early classification across four crop datasets in Europe and Africa used class-labeled reference data and a learned stopping decision, retaining competitive accuracy while reducing the amount of seasonal data used [23].

In contrast, the present Jilin 2019 study uses four monthly Sentinel 1 and Sentinel 2 slots with selected observation subsets and three public crop maps assembled as a pseudo reference. The July and August student reaches 0.792 OA and 0.742 Macro F1 in agreement with this constructed reference. STLRNet keeps a fixed four-month interface, does not reconstruct unavailable observations, and combines explicit month availability with multi-source supervision. Differences in region, period, observation density, class system, reference source, and metric definition prevent direct numerical ranking; the external evidence shows that incomplete and early sequences can support crop mapping under their own references, whereas the present evidence is limited to map-derived agreement in one region and year.

### 4.1. Interpretation of the Main Results

Under the fixed July and August setting, the STLRNet student achieves the highest agreement among the sparse models, reaching 0.792 OA, 0.772 AA, 0.647 Kappa, 0.742 Macro F1, and 0.596 mIoU. The Transformer is the strongest baseline, whereas U-Net, DeepLabV3+, and TempCNN remain lower across all metrics. This ranking is consistent with the combined use of masked temporal aggregation, confidence-weighted pseudo supervision, and teacher–student distillation, but it represents relative agreement under a common constructed reference rather than verified crop accuracy.

This ranking concerns the complete systems summarized in Table 2. The selected baselines share the input months, split, pseudo reference, and checkpoint rule with the student, but they do not receive full-temporal teacher logits or bottleneck features and do not use prototype compactness. The observed differences therefore support the tested STLRNet system as a whole and do not establish that its network architecture alone accounts for the performance gap.

Month sensitivity in Table 4 shows a consistent increase in agreement as temporal coverage expands. July yields 0.682 OA and 0.567 mIoU, while August yields 0.697 OA and 0.571 mIoU. Their paired setting reaches 0.792 OA and 0.596 mIoU; June to August reaches 0.807 OA and 0.633 mIoU, July to September reaches 0.813 OA and 0.657 mIoU, and the complete June-to-September sequence reaches 0.821 OA and 0.671 mIoU. Additional phenological context is a plausible explanation for this pattern, but the evidence is limited to one region, year, split, and pseudo reference.

Class-wise metrics in Table 5 show that the reported performance varies by category. Rice has the highest recall (0.941), whereas maize has the highest precision (0.896), F1 (0.816), and IoU (0.689). Soybean has the lowest precision (0.548), recall (0.673), F1 (0.604), and IoU (0.433). These metrics characterize agreement with the constructed pseudo reference and cannot determine which source labels are correct without independent field validation.

Supervision results in Table 6 distinguish restricted evaluation from broader all-region supervision. High-confidence regions reach 0.824 OA, 0.815 Macro F1, and 0.712 mIoU, whereas unweighted hard labels obtain 0.721, 0.693, and 0.532. Weighting raises these values to 0.732, 0.706, and 0.547, and soft pseudo labels reach 0.751, 0.718, and 0.562; conflict OA and Macro F1 change from 0.617 and 0.581 for hard labels to 0.623 and 0.592 with weighting, and to 0.638 and 0.608 with soft labels. Because restricted rows exclude ambiguous pixels, they represent an easier evaluation population and are not directly comparable with the complete student.

Component ablation in Table 7 shows a consistent ordering across OA, Kappa, and mIoU. Relative to the complete student with 0.792 OA, 0.647 Kappa, and 0.596 mIoU, removing feature distillation produces the largest reductions of 0.080, 0.034, and 0.098, respectively. Removing logit distillation gives the next largest reductions of 0.051, 0.018, and 0.055. Omitting compactness loss and matching the teacher width have smaller effects, with Kappa decreasing by 0.015 and 0.010, respectively. The combined design is therefore useful under the tested configuration, although these comparisons do not isolate all optimization interactions or establish that every component will remain necessary on other datasets.

The three regularization terms operate at different levels of the learning process. Logit distillation transfers the teacher class distribution, feature distillation constrains the student bottleneck, and prototype compactness promotes class-wise concentration within the student representation. Their ablations indicate complementary roles under the tested configuration, but the single-run design does not establish a stable causal ranking among these mechanisms. In particular, the larger observed reduction after removing feature distillation should not be interpreted as proof that channel truncation is optimal or that feature transfer will dominate in other regions and years.

### 4.2. Computational Interpretation

Efficiency measurements show that the student reduces parameters from 780,549 to 443,141, GFLOPs from 19.106 to 11.309, inference time from 5.82 to 4.61 ms, and memory from 5091.5 to 3946.3 MB. This reduction is accompanied by lower agreement than the full-temporal teacher, with OA changing from 0.883 to 0.792 and Macro F1 from 0.875 to 0.742. Exported computation reflects channel width and the complete implementation rather than the isolated cost of active month processing.

The practical distinction from imputation-based methods is that STLRNet does not reconstruct unavailable observations. It retains a fixed month axis and suppresses unavailable evidence during temporal aggregation. The distinction from early classification methods is that the present study evaluates selected month subsets rather than learning an adaptive decision time. Its main practical value is therefore the coordinated use of explicit month availability, map conflict information, complete sequence teacher guidance, and a compact deployment model. These advantages concern workflow integration under the tested setting and do not establish superiority over specialized imputation or early classification methods under their own protocols.

### 4.3. Limitations and Scope

Several limitations constrain the scope of the findings. Grid identifier splitting can place the same tile identifier in more than one subset, the experiment covers only one province and year, three public crop maps provide all supervision, the main experimental comparisons use one random seed, and source weights remain heuristic. Without independent field or parcel labels, every numerical result measures consistency with the assembled pseudo reference rather than verified crop identity or spatially independent generalization.

The main experimental comparisons use a fixed random seed of 42, providing a reproducible reference configuration under the specified training protocol. However, a single run cannot characterize variability arising from parameter initialization, mini-batch ordering, and random augmentation. Consequently, the magnitude of an observed improvement, and particularly the ranking of methods with small performance differences, may change across repeated runs. Using the same seed across methods does not establish that their performance differences are stable.

As a supplementary assessment, the STLRNet student was evaluated using random seeds 42, 123, and 2024 with the July and August observations, while keeping the data split, hyperparameters, training procedure, and checkpoint selection rule unchanged. The mean and sample standard deviation across these three runs were 0.792±0.002 for OA, 0.743±0.005 for Macro F1, and 0.597±0.004 for mIoU. The observed variation provides limited evidence of repeatability for the proposed student under this fixed configuration. However, corresponding multiple-seed results are not reported for the baselines and ablation settings, so this assessment does not establish the statistical significance or stability of the improvements over those alternatives. Three runs also provide a limited estimate of training variability and do not assess sensitivity to alternative data splits or generalization across regions and years.

Cross-year and cross-region transfer lies outside the current evaluation scope. Phenological shifts, cultivation changes, sensor conditions, and feature distribution differences may reduce performance outside the study setting. The evidence is therefore limited to the Jilin 2019 pseudo reference, the fixed grid split, the evaluated training configurations, and the heuristic source weights used here.

## 5. Conclusions

In this paper, we developed a confidence-weighted sparse-temporal crop mapping framework for incomplete seasonal observations and disagreement among multiple public crop maps. Harmonized source labels provide hard targets, heuristic weights, soft distributions, and conflict region codes, while masked monthly aggregation, teacher–student distillation, and a compact student architecture integrate temporal and supervision uncertainty. Under the fixed July and August comparison in the Jilin 2019 case study, the STLRNet student reaches 0.792 OA, 0.772 AA, 0.647 Kappa, 0.742 Macro F1, and 0.596 mIoU, and OA increases from 0.682 for July alone to 0.821 for the complete June-to-September sequence. Soft pseudo labels improve OA from 0.721 to 0.751 and conflict OA from 0.617 to 0.638 relative to unweighted hard labels, while the component analysis records the largest single ablation reductions when feature or logit distillation is removed. These observations describe the complete tested systems and do not isolate an architecture-only advantage or establish stable effects across runs. Relative to the full-temporal teacher, the student reduces parameters, exported computation, inference time, and memory at the cost of lower agreement.

## Figures and Tables

**Table 1:** Data sources and label harmonization for the constructed pseudo reference. The table lists the source year, native grid, raw class coding, and role in supervision construction.

Data Source	Year	Native Grid	Raw Classes	Role
Majorcrop	2019	about 10 m	1 rice, 2 maize, 3 soybean, 4 other	primary source
Crop10m	2019	about 10 m	0 rice, 1 maize, 2 soybean, 3 other land	secondary source
NEcrops30m	2019	about 30 m	1 rice, 2 maize, 3 soybean, 4 other	coarse agreement source

**Table 2:** Training information used for the main model comparison. The table summarizes input months, pseudo-label losses, and model-specific regularization.

Model	Input Months	Pseudo-Label Losses	Additional Regularization
U Net	July and August	Weighted cross-entropy, weighted Dice, soft target	Class weighting and geometric augmentation
DeepLabV3 plus	July and August	Weighted cross-entropy, weighted Dice, soft target	Class weighting and geometric augmentation
TempCNN	July and August	Weighted cross-entropy, weighted Dice, soft target	Class weighting and geometric augmentation
Transformer	July and August	Weighted cross-entropy, weighted Dice, soft target	Class weighting and geometric augmentation
STLRNet Student	July and August	Weighted cross-entropy, weighted Dice, soft target	Prototype compactness, class weighting, and geometric augmentation
STLRNet Teacher	June through September	Weighted cross-entropy, weighted Dice, soft target on agreement-focused supervision	Prototype compactness, class weighting, and geometric augmentation

**Table 3:** Main model comparison under the fixed July and August grid-based evaluation. Values report OA, AA, Kappa, Macro F1, and mIoU against the constructed pseudo reference.

Method	OA	AA	Kappa	Macro F1	mIoU
U-Net	0.682	0.591	0.533	0.634	0.473
DeepLabV3+	0.693	0.604	0.542	0.639	0.487
TempCNN	0.698	0.611	0.550	0.643	0.507
Transformer	0.713	0.638	0.573	0.673	0.522
STLRNet Student	0.792	0.772	0.647	0.742	0.596

**Table 4:** Student performance across input month settings under the fixed grid-based split and common label construction. Values report the main agreement metrics.

Input Months	OA	AA	Macro F1	mIoU
July	0.682	0.606	0.631	0.567
August	0.697	0.615	0.659	0.571
July to August	0.792	0.772	0.742	0.596
June to August	0.807	0.786	0.774	0.633
July to September	0.813	0.803	0.792	0.657
June to September	0.821	0.814	0.802	0.671

**Table 5:** Class-wise precision, recall, F1, and IoU for the July and August student run against the constructed pseudo reference.

Class	Precision	Recall	F1	IoU
Rice	0.617	0.941	0.745	0.594
Maize	0.896	0.749	0.816	0.689
Soybean	0.548	0.673	0.604	0.433
Other	0.895	0.725	0.801	0.668

**Table 6:** Supervision and label ablation results. Values report overall and conflict region agreement with the constructed pseudo reference.

Supervision Setting	OA	Macro F1	mIoU	Conflict OA	Conflict F1
Majorcrop only	0.821	0.812	0.694	-	-
Crop10m only	0.806	0.787	0.668	-	-
NEcrops30m only	0.793	0.767	0.626	-	-
High-confidence regions	0.824	0.815	0.712	-	-
Hard labels without weights	0.721	0.693	0.532	0.617	0.581
Weighted pseudo labels	0.732	0.706	0.547	0.623	0.592
Soft pseudo labels	0.751	0.718	0.562	0.638	0.608
STLRNet Student	0.792	0.742	0.596	0.674	0.635

**Table 7:** Component ablation results relative to the full STLRNet student model. Rows report the full student or a setting that removes or matches one component. OA, AA, Kappa, Macro F1, and mIoU quantify agreement with the constructed pseudo reference.

Model Ablation	OA	AA	Kappa	Macro F1	mIoU
STLRNet Student	0.792	0.772	0.647	0.742	0.596
Without logit distillation	0.741	0.723	0.629	0.701	0.541
Without feature distillation	0.712	0.683	0.613	0.658	0.498
Without compactness loss	0.748	0.731	0.632	0.712	0.554
Equal teacher–student width	0.763	0.741	0.637	0.719	0.564

**Table 8:** Computational efficiency comparison between the exported STLRNet teacher and student. Parameter count, GFLOPs, inference time, memory, OA, and Macro F1 are reported for agreement with map-derived labels.

Model	Params	GFLOPs	Inference ms	Memory MB	OA	Macro F1
STLRNet Teacher	780,549	19.106	5.82	5091.5	0.883	0.875
STLRNet Student	443,141	11.309	4.61	3946.3	0.792	0.742

## Data Availability

Sentinel 1 and Sentinel 2 observations used in this study are publicly available from the Copernicus programme. Public crop map products should be obtained from their original providers. Source code, configuration files, fixed split indexes, pseudo-label construction rules, and derived experiment records used in this study are available from the corresponding author upon reasonable request. Trained checkpoints can also be provided subject to storage and institutional conditions.

## Abbreviations

Abbreviations used in this manuscript are listed below:

STLRNet	Sparse-Temporal Label-Robust Network
SAR	Synthetic Aperture Radar
NDVI	Normalized Difference Vegetation Index
KD	Knowledge Distillation
OA	Overall Accuracy
Macro F1	Macro-Averaged F1 Score
mIoU	Mean Intersection over Union
